# Nucleolar Organization and Functions in Health and Disease

**DOI:** 10.3390/cells9030526

**Published:** 2020-02-25

**Authors:** Ursula Stochaj, Stephanie C. Weber

**Affiliations:** 1 Department of Physiology, McGill University, Montreal, QC H3G 1Y6, Canada; 2 Department of Biology, McGill University, Montreal, QC H3A 1B1, Canada

**Keywords:** nucleolus, liquid-liquid phase separation, ribosome biogenesis, chromatin organization, stress, aging, ribosomopathies, cancer

## Abstract

The nucleolus is a prominent, membraneless compartment found within the nucleus of eukaryotic cells. It forms around ribosomal RNA (rRNA) genes, where it coordinates the transcription, processing, and packaging of rRNA to produce ribosomal subunits. Recent efforts to characterize the biophysical properties of the nucleolus have transformed our understanding of the assembly and organization of this dynamic compartment. Indeed, soluble macromolecules condense from the nucleoplasm to form nucleoli through a process called liquid–liquid phase separation. Individual nucleolar components rapidly exchange with the nucleoplasm and separate within the nucleolus itself to form distinct subcompartments. In addition to its essential role in ribosome biogenesis, the nucleolus regulates many aspects of cell physiology, including genome organization, stress responses, senescence and lifespan. Consequently, the nucleolus is implicated in several human diseases, such as Hutchinson–Gilford progeria syndrome, Diamond–Blackfan anemia, and various forms of cancer. This Special Issue highlights new insights into the physical and molecular mechanisms that control the architecture and diverse functions of the nucleolus, and how they break down in disease.

## 1. Functional Organization of the Nucleolus

### 1.1. Subcompartmentalization and Ribosome Biogenesis

Correll and colleagues present a comprehensive review of the structure and function of the human nucleolus [1]. Integrating classic ultrastructural and biochemical evidence with recent advances from modern biophysical techniques and cryoEM, these authors describe a compartment that remains intact despite the continuous exchange of components and rapid flux of ribosome intermediates. The human nucleolus is nucleated at rDNA sites, where weak and transient interactions among nascent rRNA and the intrinsically disordered regions of hundreds of proteinaceous assembly factors give rise to three liquid phases that are mutually immiscible. Differences in surface tension generate a core-shell architecture, with the fibrillar center (FC) surrounded by the dense fibrillar component (DFC), which are both encompassed by the granular component (GC). The relative positions of these subcompartments facilitate efficient ribosome biogenesis from the inside out: rRNA transcripts are produced in the FC; spliced, modified and folded in the DFC; and assembled with r-proteins in the GC. In addition to spatiotemporally separating the steps of ribosome biogenesis, the unique material properties of each subcompartment may also contribute to these biogenesis activities themselves.

To address this question, efforts are underway to develop new methods, including proximity labeling [2] and optogenetic tools [3]. In this Special Issue, Kim et al. [4] introduce optical diffraction tomography (ODT) as another technique to examine the physicochemical properties of the nucleolus. ODT is a label-free interferometric technique capable of high-speed, three-dimensional imaging of live cells and tissues. Kim et al. demonstrate that ODT can detect differences in the refractive indices of nucleolar subcompartments in human cells. Moreover, it can be used to monitor changes in nucleolar structure in response to chemical perturbation, such as the formation of nucleolar caps following treatment with the transcription inhibitor actinomycin D [5]. 

Complementing work in human cells, this Special Issue also highlights progress in characterizing the nucleolus in non-traditional model organisms. Martinez-Calvillo et al. [6] discuss the unusual features of ribosome biogenesis in trypanosomes. In contrast to the tripartite organization found in humans, the trypanosome nucleolus has a bipartite structure that seemingly lacks a FC. The apparent absence of this core subcompartment may be due to the relatively small amount of rDNA in these protozoans. Indeed, *Leishmania major* and *Trypanosoma brucei* carry only one to two dozen copies of rRNA genes, compared to several hundred in the human genome. In addition to a low copy number, the 28S rRNA gene is fragmented in trypanosomes, requiring additional processing steps. Thus, an alternative explanation for the bipartite structure could be an enlargement of the DFC (at the expense of the FC) to accommodate the extra splicing and modifications necessary to assemble the unique trypanosomatid 60S ribosomal subunit.

O’Day tackles the even more unusual structure of the nucleolus in *Dictyostelium discoideum* [7]. Although no subcompartments are observed by electron microscopy, immunofluorescence recently revealed at least six distinct protein localization patterns in the nucleolus. Some of these patterns likely arise through interactions with the nuclear envelope, as nucleoli are located in several patches at the nuclear periphery in *Dictyostelium*. How these putative subcompartments correspond to the well-characterized FC, DFC and GC is unclear, particularly given its seemingly inverted architecture: rDNA is located in a ring around the periphery of the nucleolus, rather than at its core. The *Dictyostelium* proteome has an unusually high abundance of prion-like domains, which drive phase separation and/or aggregation in other organisms but remain soluble in *Dictyostelium* [8]. It will be interesting to dissect the mechanisms by which nucleolar proteins condense and self-organize within this unique proteostasis landscape. Future investigations of nucleoli in trypanosomes, *Dictyostelium* and other non-model organisms promise to shed new light on the structure-function relationships in this essential compartment.

Nucleoli associate with the nuclear membrane in *Dictyostelium* [7] and *S. cerevisiae* [9]. Interestingly, components of the nuclear envelope modulate the properties of nucleoli [10,11]. Essawy et al. [12] examined emerin, a protein located in nuclear membranes. They show that some emerin mutants compromise the cellular response to mechanical stress and affect the linker of the nucleoskeleton and cytoskeleton (LINC) complex. These studies set the stage to determine how emerin and other proteins of the mammalian nuclear envelope impinge on nucleoli. 

Two papers in this Special Issue focus on the structure-function relationship of individual proteins. In a primary research article, Duan and colleagues [13] examine the localization and activity of poly(A)-specific ribonuclease (PARN). PARN is a multi-domain protein with an intrinsically disordered C-terminal domain. Through a series of targeted deletions, the authors identify nuclear and nucleolar localization sequences within this domain, as well as binding sites for the regulators CBP80 and CstF-50. Phosphomimetic mutations reveal that unmodified PARN associates with CBP80, which inhibits its deadenylase activity in the nucleolus, while phosphorylated PARN recruits CstF-50, which activates it. Phosphorylation is triggered by DNA damage, leading to changes in the processing of small RNAs that may protect cells against genotoxic stress.

Sleiman and Dragon [14] review the literature on NAT10, a ribosome assembly factor known as Kre33 in yeast. NAT10 contains both an N-acetyltransferase domain and a helicase domain, as well as nuclear and nucleolar localization sequences near its C-terminus. It acetylates multiple substrates, including pre-18S rRNA, tRNAs and mRNAs. In addition, the autoacetylation of NAT10 is required for the activation of rRNA transcription. Interestingly, inhibition of NAT10 restores nuclear shape in the human laminopathy Hutchinson–Gilford progeria syndrome (HGPS), presenting a potential therapeutic target (see Section 2.2. for more details). Together, PARN and NAT10 underscore the importance of post-translational modifications in regulating both the structure and function of nucleolar proteins.

### 1.2. Genome Organization and Regulation of Gene Expression 

Bersaglieri and Santoro [15] discuss how the epigenetic state of rDNA regulates not only ribosome biogenesis, but also the spatial organization and transcriptional activity of the genome more globally. rDNA exists in three distinct chromatin states: active, inactive and silent. Active rRNA genes are nucleosome-free and bound by the upstream binding factor (UBF) and the transcription intermediary factor B (TIF-1B), which initiate transcription by RNA polymerase I. In contrast, the promoters of silent rRNA genes are methylated and their coding regions are packed by nucleosomes with repressive histone marks. During differentiation, the nucleosome remodeling complex (NoRC) recruits DNA methyltransferases to active rRNA genes and establishes silent heterochromatin at the nucleolus. These now-silenced rRNA genes are important for genome stability and form hubs around which non-ribosomal DNA organizes into nucleolus-associated domains (NADs). Though not contiguous with rDNA, NADs are also enriched for repressive marks and remain transcriptionally silent. The spatial proximity of the nucleolus and NADs may be driven by the mechanical exclusion of intervening euchromatin and the attraction of condensed genomic loci [16]. 

In support of this hypothesis, Legartova et al. [17] find that heterochromatin protein 1 (HP1) forms foci near the nucleolus in human cells. These foci are reminiscent of constitutive heterochromatin domains in *Drosophila*, which assemble through phase separation of HP1α [18]. Interestingly, Legartova and colleagues observe isoform-specific behavior in response to DNA damage, suggesting that HP1α foci may differ in their mechanical properties. Moreover, exclusion of TIF-1B from nucleolus-proximal HP1β and HP1γ foci point to a potential role in transcriptional silencing of NADs. 

## 2. Nucleoli—Key Players for Stress Responses, Aging and Human Health 

### 2.1. Stress, Aging, and Longevity 

The critical role of nucleoli for stress responses is firmly established [19,20,21]. Pirogov et al. [22] discuss how long noncoding RNAs (lncRNAs, defined as RNAs with more than 200 nucleotides) regulate ribosome production. Various lncRNAs participate in these regulatory events. Their coding region is located within or outside of rDNA clusters. LncRNAs are transcribed by different RNA polymerases, either in nucleoli or the surrounding nucleoplasm. When associated with nucleoli, they function in stress and disease-specific fashion. 

A set of lncRNA-based processes adjusts pre-rRNA synthesis to the physiological and environmental conditions of cells. Two major mechanisms are involved, lncRNA-mediated control of rDNA transcription and protein sequestration in nucleoli. To promote stress survival, nucleolar lncRNAs retain selected proteins and limit rRNA synthesis. To this end, lncRNAs operate through multiple pathways, such as nucleosome remodeling and epigenetic chromatin modifications [22]. Overall, nucleolar lncRNAs help to reduce energy-intensive ribosome biogenesis under unfavorable conditions. Intriguingly, nucleolar lncRNAs may also participate in local phase separation.

Matos-Perdomo and Machín [9] present studies in the model organism *Saccharomyces cerevisiae* that unraveled some of the puzzles related to cellular stress responses. They describe the nucleolar features shared by budding yeast and mammalian cells as well as the unique properties of nucleoli in *S. cerevisiae*. Mutant and other analyses identified yeast factors that regulate the transcription of rDNA genes, homologous recombination of rDNA, rDNA association with the nuclear envelope, pre-rRNA processing and other aspects of ribosome biogenesis. Of particular interest in the context of yeast aging is the formation of extrachromosomal rDNA rings (ECRs). The production of ECRs causes nucleolar dysfunction and promotes aging in *S. cerevisiae*. 

Many principles determining the organization and function of yeast and mammalian nucleoli are shared. For example, Matos-Perdomo and Machín [9] portray the silencing complexes and key signaling routes that impinge on nucleolar morphology and individual steps of ribosome biogenesis. In yeast and mammals, the TOR (target of rapamycin) complex and sirtuins are central regulators of nucleolar morphology and function. Importantly, they also control lifespan and are druggable targets (see Section 2.2). 

Meriem et al. [23] examined the hyperosmotic stress response in *S. cerevisiae* using microfluidics and single-cell analyses. To this end, they studied the stress-responsive *STL1* promoter. When exposed to two consecutive rounds of hyperosmotic stress, most yeast cells displayed memory of the first stress event. Yet, within the population, memory varied among single cells. Interestingly, de novo protein synthesis during stress exposure was not a pre-requisite for the memory effect. Instead, the chromatin environment was critical for cells to exhibit memory. It will be interesting in the future to determine whether and how memory effects are shaping the nucleolar stress response.

Persistent oxidative stress is a hallmark of cellular senescence and aging [24]. Thus, it is not surprising that nucleoli exhibit aging-associated changes. Details of these processes are now being uncovered. Recent studies in vitro and in vivo provided new insights that further substantiate the importance of nucleoli for senescence and aging at the cellular or organismal level. These studies are being discussed in several reviews published in *Cells*, including this Special Issue and previous publications [1,9,22,25].

While nucleolar properties are affected by aging, it is much more difficult to establish a causative link. For example, does the derailment of the nucleolar organization or function cause physiological alterations that are related to aging? Indeed, for budding yeast several lines of evidence support the idea that nucleolar dysfunction instigates aging (see above). Insights supporting this concept are now beginning to emerge for mammalian cells as well, in particular through the research on childhood and other progerias. 

Phan et al. [26] discuss the work on HGPS, Cockayne Syndrome (CS) and related segmental progeroid syndromes that puts the nucleolus in the spotlight of aging research. These model systems exhibit some changes associated with normal aging, but do not display the full range of aging-related physiological decline. Nevertheless, experimental evidence supports that some premature aging syndromes are driven by dysfunctional nucleoli. For example, secondary DNA structures, which are mostly present in rDNA, cannot be properly resolved in CS cells. Ultimately, this could result in premature aging, as it is observed for CS patients [26]. 

A more general hypothesis identifies nucleoli as instigators of premature aging for CS. In this scenario, malfunctional rDNA transcription generates ribosomes that are unable of high-quality performance. These ribosomes synthesize defective polypeptides, including ribosomal proteins. A vicious circle ensues. Subsequently produced ribosomes will have even lower quality, and misfolded proteins will accumulate [26]. This model links the improper organization of rDNA in CS to impaired ribosome assembly, faulty translation, and, in the long run, a derailment of proteostasis. 

Corell et al. [1] point out an interesting conundrum, related to the nucleolus in aging cells. In HGPS patient cells rDNA transcription is elevated. At the same time, nucleolar size, ribosome production and protein synthesis increase. Collectively, the upregulation of these energy-intensive processes may deplete cellular energy levels and promote organismal aging. By contrast, “normal” physiological aging is accompanied by reduced rDNA transcription, nucleolar size, ribosome biogenesis, and translation. It is not apparent at the moment how these contradictions can be easily reconciled.

Interestingly, the methylation of CpG motifs in rDNA highly correlates with the age of experimental mice [1]. This “clock” slows down when lifespan is extended by calorie restriction. Whether CpG methylation in rDNA can provide a reliable readout for human aging is an important question for future research.

### 2.2. Ribosomopathies and Cancer

Corell et al. and Penzo et al. [1,27] discuss several links that connect nucleolar biogenesis to disease. Thus, the failure to assemble ribosomes properly can lead to a spectrum of disorders, collectively called ribosomopathies [28]. In general, these congenital disorders arise through haploinsufficiency, with loss-of-function for one copy of a gene required for ribosome production. Such disease-causing haploinsufficiency has been observed for ribosomal proteins and other components of the ribosome assembly pathway. Interestingly, these mutations often have tissue-specific effects; several models are described to explain the tissue-specificity [1]. 

So far, ribosomopathies can be separated into two groups (i) inherited bone marrow failure syndrome (IBMFS; including Diamond-Blackfan anemia, Shwachman-Diamond syndrome, Dyskeratosis congenita) and (ii) Treacher Collins Syndrome. Ribosomopathies of the IBMFS group predispose patients to cancer. Corell et al. and Penzo et al. [1,27] illustrate possible mechanisms underlying this cancer predisposition. The authors also review how specific oncogenes and tumor suppressors partake in these mechanisms. 

Taken together, nucleolar activities determine the assembly rates of ribosomes. They also conduct essential quality control steps during ribosome biogenesis. Several independent studies indicate that alterations in ribosome quality and quantity lead to cancer.

As described by multiple contributions to this Special Issue, proper nucleolar function is essential to human health. The impact of nucleoli on “The Ribosome Biogenesis—Cancer Connection” is particularly well-established. It is the focus of Penzo et al. [27], while Carotenuto et al. [29] discuss in detail how nucleoli can be targeted for cancer therapy. 

Nucleolar size and the silver-staining of nucleolar organizer regions (NORs) during interphase have long been recognized as a useful tool to identify nucleolar changes that are relevant to tumor pathology. Signals obtained by AgNOR-staining correlate with ribosome biogenesis. This method has diagnostic value and uncovers nucleolar hypertrophy. However, the authors caution that the sizes of nucleoli overlap between malignant and benign lesions [27]. Accordingly, nucleolar size helps to assess cancer pathology, but needs to be evaluated in the context of other diagnostic and prognostic markers. 

To provide in-depth information on the ribosome biogenesis cancer link, Penzo et al. [27] illustrate the molecular processes that are triggered by the inhibition of ribosome production. Key players, including p53 (tumor suppressor protein), pRB (retinoblastoma protein) and Myc (transcription factors encoded by proto-oncogenes), and their specific impact on nucleolar function are discussed. The authors emphasize the importance of chronic inflammation for cancer development and progression. Chronic inflammation is characterized by increased levels of IL6, which prompts IL6-dependent upregulation of ribosome biogenesis. This, in turn, promotes nucleolar hypertrophy and cancer cell proliferation. 

Carotenuto et al. [29] concentrate on recent developments in cancer therapy that aim at nucleoli. The authors focus on druggable targets and portray approaches that interfere with cell proliferation. Current research explores two overall strategies: (i) targeting individual factors required for ribosome biogenesis, and (ii) modulating signaling events pertinent to ribosome production and tumor malignancy. 

Different compounds have been evaluated for their ability to interfere with ribosome biogenesis. The tested agents may interact directly with nucleolar proteins or alter nucleolar functions through various pathways. Carotenuto et al. [29] discuss selected drugs and their mode of action. Other possible means to target nucleoli, such as nanoparticles, are also presented. 

RNA polymerase I and interacting factors provide prime targets for cancer therapy. Drug development has also been successful for diverse regulators of nucleolar activities. Specific examples are signaling routes that involve Myc, Ras/ERK, mTOR, Akt, PTEN, pRB, p14^ARF^, and especially p53. 

Nucleolar stress pathways operate in a p53-dependent or p53-independent fashion. Given that p53 mutations are linked to a large number of human cancers, this tumor suppressor has been widely evaluated for therapeutic intervention. Carotenuto et al. [29] present an up-to-date overview of different agents that alter nucleolar function through p53. To date, several clinical trials are assessing drugs that have been designed to interfere specifically with nucleolar activities [30]. On the other hand, a large number of compounds tested or FDA-improved for cancer treatment alter nucleolar organization and function [29,30].

## 3. Outlook—Open Questions

The understanding of nucleolar biology has come far since the early description of a “corps oviforme” in 1781 (Fontana, see [9]). Conceptual advances related to the assembly of nucleoli through phase separation and their contributions to human health and disease have propelled the field to new research avenues. Recent insights led to a better appreciation of nucleoli as key players for cellular physiology, and possible drivers of aging or disease. Yet, publications included in this Special Issue point to numerous open questions that remain to be answered.

Future topics to be addressed include fundamental aspects of nucleolar functions and the translation of current knowledge into clinical applications. Box 1 depicts some of the areas that require further research.

Box 1 **Nucleolar organization and functions in health and disease—open questions. Topics that remain poorly understood in the field of nucleolar biology or relevant to knowledge translation are specified. The list is not comprehensive; more details can be found in the publications cited in this Editorial.**

Nucleoli are organized into subcompartments with different biological activities. How do the material properties of each sub-phase impact its function? How are ribosome intermediates transported vectorially from one phase to the next?

How do the non-canonical structures of nucleoli in trypanosomes and *Dictyostelium* affect ribosome biogenesis in these evolutionarily divergent organisms?

Can we identify sequence features that define each nucleolar subcompartment? Can such features be used to predict protein interaction networks, material properties and biochemical activities?

What are the physical and molecular mechanisms through which the nucleolus regulates genome organization and transcriptional activity?

Stress, cellular quiescence and tumorigenesis are linked to the nucleolar association of lncRNAs and other RNAs. Not all of the mechanisms have been defined at the molecular level. Is the action of different regulatory RNAs coordinated? How do these RNA-based pathways communicate with other cell signaling mechanisms?

How do cellular memory effects impinge on nucleoli and the nucleolar stress response in particular?

Can the nucleolus serve as a “clock” for cell, organ, or organismal aging? If yes, which cells or organs provide information that is most meaningful to assess organismal aging?

There is a large body of information on ribosome assembly. By contrast, knowledge pertinent to other nucleolar activities is comparably sparse. How are these other nucleolar functions contributing to cellular homeostasis? Which activities are relevant to human health?

How does nucleolar stress affect different cell types, developmental stages and disease states?

Are cell types and tissue-specific differences of nucleoli contributing to the diversity of ribosome populations? Are nucleoli on top of the chain that controls which mRNAs will be translated? If yes, can nucleolar activities be altered to modulate the translational profile of a cell?

Nucleolar changes are associated with segmental progeroid disorders. To which extent are these pathology-driven changes relevant to “normal” aging? Which nucleolar alterations are specific to progeroid disorders? Can they be exploited for patient treatment?

If nucleolar properties drive aging—are nucleoli realistic targets to promote healthy aging and longevity?

Nucleoli contribute to the organization of the nucleoplasm and, through the nucleo- and cytoskeleton, may also communicate with the cytoplasm. What are the molecular players and mechanisms mediating this communication?

How can disease-associated qualitative and quantitative changes of nucleoli and ribosomes be exploited for therapy? What strategies are successful for different types of cancer, ribosomopathies, viral infections or other conditions?
